# Opposing Subjective Temporal Experiences in Response to Unpredictable and Predictable Fear-Relevant Stimuli

**DOI:** 10.3389/fpsyg.2018.00360

**Published:** 2018-03-21

**Authors:** Qian Cui, Ke Zhao, Yu-Hsin Chen, Weiqi Zheng, Xiaolan Fu

**Affiliations:** ^1^State Key Laboratory of Brain and Cognitive Science, Institute of Psychology, Chinese Academy of Sciences, Beijing, China; ^2^Department of Psychology, University of Chinese Academy of Sciences, Beijing, China; ^3^Key Laboratory of Mental Health, Institute of Psychology, Chinese Academy of Sciences, Beijing, China; ^4^College of Teacher Education, Wenzhou University, Wenzhou, China

**Keywords:** time perception, fear, emotion, expectancy, unpredictability

## Abstract

Previous studies have found that the durations of fear-relevant stimuli were overestimated compared to those of neutral stimuli, even when the fear-relevant stimuli were only anticipated. The current study aimed to investigate the effect of the predictability of fear-relevant stimuli on sub-second temporal estimations. In Experiments 1a and 1b, a randomized design was employed to render the emotional valence of each trial unpredictable. In Experiments 2a and 2b, we incorporated a block design and a cueing paradigm, respectively, to render the emotional stimuli predictable. Compared with the neutral condition, the estimated blank interval was judged as being shorter under the unpredictable fear-relevant condition, while it was judged as being longer under the predictable fear-relevant condition. In other words, the unpredictable and predictable fear-relevant stimuli led to opposing temporal distortions. These results demonstrated that emotions modulate interval perception during different time processing stages.

## Introduction

Fear is a ubiquitous emotion that can be a reaction to a particular stimulus occurring in the present or to a possible anticipated future threat. People often report that when confronted with fearful or threatening stimuli, such as live spiders, car accidents and robberies, the passage of time appears to slow or even stop (Watts and Sharrock, 1984; Tse et al., 2004). Numerous studies have verified the modulation of the perception of time using a variety of fearful stimuli, including fearful faces (Tipples, 2008, 2011; Eberhardt et al., 2016), scenes (Angrilli et al., 1997; Grommet et al., 2011; Fayolle et al., 2015), and sounds (Droit-Volet et al., 2010). The perceived durations of fearful stimuli are often judged as longer than those of neutral stimuli (Droit-Volet and Meck, 2007).

Not only the immediate fear, the anticipated fear can also lengthen perceived time. In an early study, Hare (1963) asked participants to estimate the elapsed time between two successive clicks on a stopwatch under two conditions, i.e., in one condition, a shock followed the interval, and in the other condition, shock electrodes were removed. The results revealed that trials in which intervals were followed by a shock tended to be overestimated in comparison to trials without shock. Hare speculated that the anticipation of a shock may explain this lengthening effect. However, the two conditions compared in this experiment were not manipulated strictly because the study could not eliminate the confusion of the expectation of a shock and the actual shock. More recently, Droit-Volet et al. (2010) used two well-controlled experiments to investigate the effect of the anticipation of a threatening event on time perception. In the first experiment, the participants were asked to estimate the duration of a blue circle followed by an aversive or non-aversive sound. Based on the sign before each trial, the participants could anticipate the sound that would follow. The duration was judged as longer when the participants expected an aversive compared with a non-aversive stimulus. In the second experiment, the authors further explored the effect of the temporal localization of the aversive stimulus by presenting the aversive stimulus at the beginning or end of the probe duration. Regardless of the localization of the presented aversive stimulus, a temporal overestimation of the probe duration was observed. Moreover, the lengthening effect was greater when the expectation of the forthcoming threatening stimulus was longer. Thus, even in the absence of an aversive stimulus, the anticipation of fear also affects perceived time. The lengthening effect of aversive stimuli on subjective temporal interval estimation was also found in another recent study in which the interval between action and the subsequent negative (fear or disgust) vocalization feedback was judged longer than positive or neutral feedback (Yoshie and Haggard, 2013). The researchers considered that their results may be related to the design of the predictable valence of each trial.

However, emotional stimuli tend to be unpredictable in our rapidly changing world. For example, predators (such as snakes) often cleverly use their surroundings to camouflage themselves and strike their prey promptly from the shadows. People are often unprepared to address unpredictable stimuli. To date, whether predictable and unpredictable aversive stimuli have similar effects on the temporal perception of a blank interval remains unclear. The most popular model used to explain the time illusion is the adopted internal clock model. According to this model, the timing process consists of the following three main stages: clock, memory, and decision making. The clock stage consists of a pacemaker, a switch, and an accumulator. The pacemaker emits pulses to the accumulator, and a switch is located between the pacemaker and accumulator that controls the opening and closing of the pathway. The perception of time is determined by the number of pulses in the pacemaker-accumulator (PA) clock system (Treisman, 1963; Gibbon et al., 1984). The expectation of a fearful stimulus is associated with an increase in the arousal level, which accelerates the internal clock (Droit-Volet et al., 2010). However, in the presence of unpredictable emotional stimuli, the arousal level in response to the emotional stimuli is the same as that during the blank interval. Therefore, the mechanisms by which the unpredictable fear-relevant stimuli modulate the temporal perception of the blank interval are questionable. According to the internal clock model, the clock stage of the estimated blank interval before the presentation of the emotional stimuli should be the same because the participants do not know the type of stimuli that will be presented; thus, the estimated blank intervals should have no difference. If the perceived blank interval of fear-relevant stimuli differs from that of other emotions, the effects of emotion on timing may occur during the memory or decision making stage. Previous studies indicated that performing a concurrent task that occupies working memory resources shortens the subjective temporal estimation (Fortin et al., 1993). Negative emotional contents, such as fearful pictures, were found to affect working memory performance (Kensinger and Corkin, 2003). Therefore, fear-relevant stimuli may influence time perception by disrupting working memory processes. However, the effect of emotion on the clock and memory stages of time perception is not easily dissociated in the presence of immediate emotional stimuli. Thus, the blank interval may be a better selection to explore the effects of fear-relevant stimuli on the memory stage of time perception. If the emotional stimulus following the estimated blank interval is predictable, top-down emotional anticipation exists during the blank interval, and the modulation of the temporal estimation of the stimulus is similar to that of an immediate stimulus. However, if the estimated blank interval is not followed by an explicit expected stimulus as in the unpredictable emotional condition, the separate roles of the clock and memory stages in emotional temporal distortions can be derived. We hypothesized that unpredictable fear-relevant stimuli may influence time perception by disrupting the process in which the pulses in working memory are transmitted to reference memory.

The purpose of the present study was to examine the effect of expectancy on fear-relevant temporal distortions. Specifically, we aimed to investigate the effects of unpredictable fearful stimuli on the perceived duration of the estimated blank interval. Moreover, we aimed to determine the effect of the predictability of a fear-relevant stimulus on temporal distortion by comparing the effects of unpredictable and predictable fearful stimuli on subjective estimated blank interval. Therefore, we manipulated the predictability of emotional stimuli. The happy emotion was included in the experiments and compared to the neutral and fearful emotions. In Experiments 1a and 1b, a randomized design was employed to render the emotional valence of each trial unpredictable. In Experiments 2a and 2b, the valence of the emotional stimuli was predictable via a block design and cue-induced design, respectively.

## Experiment 1

### Methods

#### Participants

Twenty-six college students (mean age = 21.85 years, *SD* = 2.68, including 14 females) participated in Experiment 1a. 18 college students (mean age = 21.61 years, *SD* = 2.17, including 17 females) participated in Experiment 1b. All participants were right-handed with normal or corrected-to-normal visual acuity and signed written informed consent. After the experiment, they were paid for their participation. These experiments were approved by the Ethics Committee of the Institute of Psychology, Chinese Academy of Sciences.

#### Materials

Twenty-four pictures obtained from the International Affective Picture System (IAPS; Lang et al., 2008) were used in Experiment 1a. According to the original rating of the valence and arousal of the IAPS image database and the emotional category data of subsets of the IAPS (Mikels et al., 2005), eight pictures that evoked isolated fear with a similar arousal level (*M* = 6.48; *SD* = 1.98), eight low-arousing pictures that were rated as neutral in valence, and eight pictures that evoked isolated happiness with a similar arousal level (*M* = 4.62; *SD* = 2.27) were used in the temporal task. To verify the arousal and valence ratings in our sample of subjects, 40 subjects who did not participate in the subsequent experiments rated the pictures using the self-assessment-manikin (SAM; Lang, 1980). One participant was removed as an outlier because the value was beyond 3 standard deviations. The fearful images received higher arousal ratings (*M* = 6.75; *SD* = 0.99), *t*(38) = 2.21, *p* < 0.05, than the happy images (*M* = 6.25, *SD* = 1.06); the fear-relevant images were judged as having a negative valence (*M* = 2.67; *SD* = 1.12), whereas the happy images were judged as having a positive valence (*M* = 7.22; *SD* = 0.85), and the difference between the images was significant: *t*(38) = -19.33, *p* < 0.001. Fifteen additional IAPS pictures were used during the rating phases. The stimuli used in Experiment 1b were also the same as those used in Experiment 1a, except for that the stimulus pictures were reduced to 21 pictures (7 pictures × 3 emotional valence) to fit the paradigm of experiment.

#### Apparatus

The participants sat in a comfortable position in front of a PC that controlled the events and recorded the responses via the psychology software product E-prime (2.0. Psychology Software Tools, Pittsburg, PA, United States). A 17-inch CRT monitor that operated at a refresh rate of 100 Hz and a resolution of 1024 × 768 pixels was used. The participants were tested individually in a dim, quiet room.

#### Procedures

The participants were seated in a dimly lit room at a distance of 60 cm from the CRT monitor. The image had an area of 14 × 12 cm and was located at the center of the display. Before the formal session of the experiment, a basic time discrimination test was conducted. The participants were instructed to estimate the blank interval duration, which lasted 300, 600, or 900 ms between the offset of a cross-fixation and the onset of a neutral picture, and a duration was presented prior to each of four trials. Only participants whose average temporal estimations for the three durations increased linearly could participate in the subsequent session.

During the initial training phase, a cross-fixation that randomly varied from 1000 to 1500 ms was presented, and after a variable blank interval, a neutral picture was shown. The actual interval between the cross-fixation and the appearance of the neutral picture was displayed on the screen at the end of each trial during the training phase. The interval ranged from 100 to 1200 ms, including 150, 280, 360, 440, 550, 640, 760, 850, 970, and 1150 ms during the first training session and 100, 200, 300, 400, 500, 600, 700, 800, 900, 1000, 1100, and 1200 ms during the second training session. Each duration appeared once. The participants were asked to discriminate the different interval durations and consider these durations during the following experiment to obtain estimations that are as accurate as possible. During this session, the participants did not have to provide any responses. The participants were instructed to not count during the interval because this could bias the scientific data. Furthermore, to teach the participants to initiate their timing by pressing a key, we conducted ten practice trials after the first training session.

**FIGURE 1:**
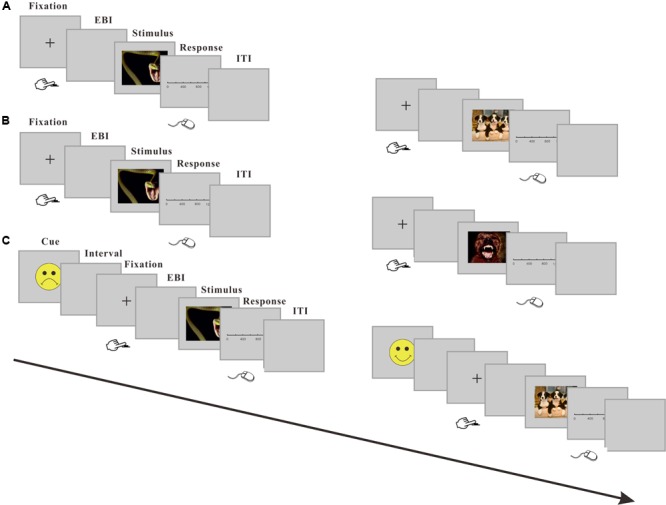
Example trial sequences from the unpredictable or predictable emotional condition. **(A)** Representation of trial sequences in the unpredictable emotional condition (Experiment 1a). One of the three valences of emotional stimuli was presented randomly in a block. The EBI (Estimated Blank Interval) was the blank interval that participants need to clock. **(B)** Representation of trial sequences in the predictable condition (Experiment 2a). The trials sequences in Experiment 2a were similar to those in Experiment 1a, except that only one of the three valences of emotional stimuli were included in a block. **(C)**. Representation of trial sequences in the predictable condition realized with cueing paradigm (Experiment 2b). The gray square trials are not shown in this figure.

During the main test phase of Experiment 1a, all three emotional valence pictures were randomly presented in a block. Therefore, the participants could not predict the emotional valence of the pictures that would appear in advance. A typical trial using this procedure is shown in **Figure 1A**. Each trial started with a cross-fixation at the center of the screen. The participants were instructed to press the key [F] on the keyboard with their left index finger. To prevent involuntary key-presses and possible mistakes, we defined the first 500 ms after the onset of the cross-fixation as an invalid phase; during this period, pressing a key would generate a warning that asked the participant to restart the current trial (e.g., Zhao et al., 2013, 2016). When the participants pressed a key after the defined invalid phase, the cross-fixation immediately disappeared. After a 610 or 640 ms blank interval, an image was presented at the center of the screen for 500 ms. Then, a response screen was shown, and the participants were asked to click on the temporal scale to indicate their estimation of the duration of the blank interval between the fixation and the image. The participants were informed to estimate the interval by clicking the mouse on the scale ranging from 0 to 1200 ms (the participants were reminded that 1000 ms is equivalent to 1 s). The participants were also told to click at any point on the scale between 0 and 1200 ms. There was no time limit to providing a response, and no feedback was given. The inter-trial interval (ITI) randomly ranged between 1200 and 2000 ms. To increase the unpredictability of the emotional stimuli, an oddball paradigm was employed. In 30% of the trials, the IAPS pictures were shown, and in the other 70% of trials, a gray square was presented. When the gray square appeared, the participants did not have to estimate the interval time, and the program automatically proceeded to the next trial. Each participant performed three blocks of 80 trials each (24 trials of pictures and 56 trials of gray squares), yielding a total of 240 trials. The participants were allowed to take a break between each block. The experiment lasted approximately 45 mins.

The procedures used in Experiment 1b were similar to those described in earlier studies (Fayolle et al., 2015). The participants underwent a training phase, followed by a testing phase. During the training phase, the participants were trained to discriminate the “short” (400 ms) from the “long” (1,600 ms) stimulus durations, which were represented by an interval duration between the presentation of a cross-fixation point and a blue square. The participants then learned to respond to the “short” and “long” durations by pressing the corresponding keys in ten trials (five trials for each standard duration) presented in random order. At the beginning of each trial, a short indication of which of the two durations would emerge next was presented on the screen for 1000 ms. Then, a cross-fixation appeared at the center of the screen for a randomly chosen duration between 1500 and 2000 ms. The offset of the cross-fixation marked the onset of the timing.

During the test phase, the participants were told that the blue square would be replaced by pictures. Each fearful, neutral and happy picture was presented twice for each of the standard (400 and 1600 ms) durations and a range of intermediate durations (600, 800, 1000, 1200, and 1400 ms). This resulted in 14 trials (7 different pictures × 2 times) for each probe duration and 98 trials (7 durations × 7 × 2) for each emotional valence. Thus, the participants completed 294 trials. In each trial, the word “ready” was first presented for 500 ms, immediately followed by a 200-ms interval, and then, the cross-fixation point was presented until the participants pressed the “F” key. The participants were required to judge whether the probe interval duration was more similar to the short or long standard interval duration by pressing the corresponding key (the “d” and “k” keys were used) with the middle fingers of their right or left hand. The response mapping of the key was counterbalanced across participants. No feedback was provided after the responses. The ITI was randomly chosen and ranged between 2000 and 2500 ms. All pictures were presented in a random order. The participants were allowed a break every 50 trials.

### Results

**FIGURE 2:**
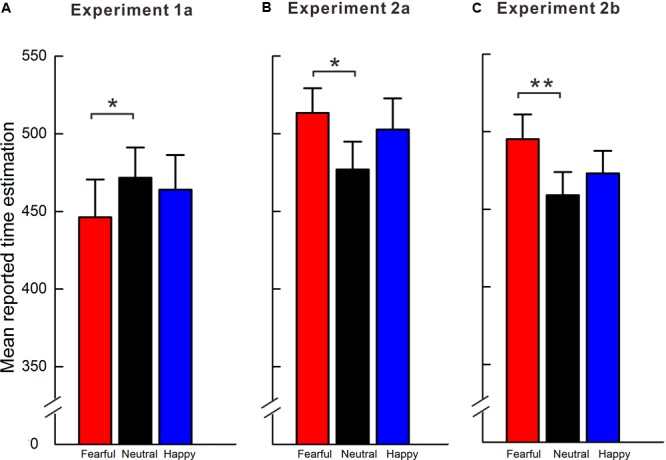
Mean reported time estimation of blank intervals under different emotional valence conditions. **(A)** In Experiment 1a, the blank interval in the unpredictable fear trial was estimated to be significantly shorter than that in the unpredictable neutral trial. **(B)** In Experiment 2a, the blank interval in the predictable fearful trial was estimated to be significantly longer than that in the predictable neutral trial using the block design. **(C)** In Experiment 2b, the lengthening effect of predictable fearful outcomes relative to predictable neutral outcomes was replicated using a cue-induced design. Error bars show ±1 SE. ^∗∗^*p* < 0.01, ^∗^*p* < 0.05.

In Experiment 1a, a repeated-measures one-way ANOVA revealed a significant difference in the estimated blank interval according to the valance of the IAPS pictures, *F*(2, 50) = 3.54, *p* < 0.05, $\eta_{p}^{2}$ = 0.12. According to the *post hoc* Bonferroni analysis, the interval under the unpredictable fearful condition was judged as significantly shorter than that under the neutral condition, *p* < 0.05, but the difference in the interval between the unpredictable happy and neutral conditions was not significant, *p* > 0.05 (see **Figure 2A**).

In Experiment 1b, the percentage results showed averaged “long” responses across all participants for each probe duration in each emotional valence (fearful vs. neutral vs. happy) trial. The fearful condition was associated with a lower percentage of long judgment than the neutral and happy conditions (see **Table 1**). A repeated-measures ANOVA of P (long) revealed a significant main effect of emotional valence, *F*(2, 34) = 3.54, *p* < 0.05, $\eta_{p}^{2}$ = 0.17, and a significant main effect of interval duration, indicating that P (long) increased as the probe duration increased, *F*(6, 102) = 191.22, *p* < 0.001, $\eta_{p}^{2}$ = 0.92. The interaction between emotional valence and interval duration was not significant, *F*(12, 204) = 1.24, *p* = 0.26. According to the *post hoc* tests (Bonferroni), the percentage of long judgment under the fearful condition was significantly lower than that under the neutral condition, *p* < 0.05. Overall, the participants judged the probe durations under the fearful condition as being shorter than those under the neutral condition in the unpredictable contexts.

**Table 1:** Proportion of long responses to probe durations in different emotional valence trials.

Valence	Probe durations						
	400 ms	600 ms	800 ms	1000 ms	1200 ms	1400 ms	1600 ms
Fearful	0.03	0.07	0.25	0.54	0.70	0.79	0.88
Neutral	0.02	0.08	0.25	0.56	0.79	0.87	0.92
Happy	0.03	0.06	0.25	0.53	0.76	0.81	0.90

*Note: The mean proportion of long responses to each probe duration of blank interval in different emotional valence trials for all participants in Experiment 1b.*

This finding was confirmed by analyses of the bisection point (BP). The BP is the point of subjective equality, i.e., the probe duration at which the participants responded “short” or “long” with equal frequency (*P*[long] = 0.5). The BP was derived from the logistic function fitted to each subject’s data. The mean BP value under the fearful, neutral and happy conditions was 1058.30 ± 143.53, 1004.67 ± 131.75, and 1039.03 ± 148.94 ms, respectively. The within-subjects repeated-measures ANOVA of the BP revealed a significant main effect of emotional valence, *F*(2, 34) = 3.38, *p* < 0.05, $\eta_{p}^{2}$ = 0.17. According to the *post hoc* tests (Bonferroni), the BP in the fearful trials was significantly larger than that in the neutral trials (*p* < 0.05), whereas the difference in the BP between the happy and neutral trials was not significant (*p* = 0.59). A larger BP indicated that the participants perceived the duration to be shorter. Thus, the participants perceived that the time passed more quickly. These results were consistent with those obtained in Experiment 1a, indicating that compared to neutral pictures, unpredictable fearful pictures shorten the interval duration. The Weber Ratio (WR) for each participant was calculated by the following expression, WR = [Interval (*P*(long) = 75% – Interval (*P*(long) = 25%)]/(2 × BP). The higher the WR value, the lower temporal sensitivity is. The mean WR value under the fearful, neutral and happy conditions was 0.14 ± 0.07, 0.12 ± 0.07, and 0.13 ± 0.07, respectively. A repeated-measures one-way ANOVA indicated that there was a significant difference in the WR value across three types of emotions, *F*(2, 34) = 4.37, *p* < 0.05, $\eta_{p}^{2}$ = 0.20. A *post hoc* tests Bonferroni analysis indicated that the WR value was significantly higher in the unpredictable fearful condition than in unpredictable neutral condition (*p* < 0.05); the WR values had no differences between happy and neutral condition (*p* > 0.05), as well as between fearful and happy condition (*p* > 0.05). This seems to indicate that the variability in interval estimation tended to be greater in unpredictable fearful condition. And this may reflect that the unexpected fearful stimuli made people shocked and less sensitive to the interval durations.

### Discussion

According to the results of Experiments 1a and 1b, under the unpredictable emotion condition, the interval between an action and its fearful outcome was estimated to be significantly shorter than that between an action and a neutral outcome. However, under the unpredictable happy condition, no difference was observed in the interval estimation from the unpredictable neutral outcome.

Under the unpredictable emotional outcome conditions, the participants could not foresee the valence of the picture until the emotional picture was presented. According to the scalar expectancy theory, during the clock stage, the perception of the blank interval between different manipulated conditions may be the same. Therefore, the differences in the reported interval durations among the three types of valence conditions should not be due to the arousal or attention level during the blank interval. This shortening effect of unpredictable fear may reflect other mechanisms underlying the emotional modulation of time. In this study, the unpredictable fear-relevant stimuli may have interfered with temporal information processing. According to previous studies, temporal durations are judged as shorter while performing an interference task. This shortening effect can be explained by the loss of pulses during the rehearsal of the temporal information in working memory or short-term memory (Wearden and Ferrara, 1993; Wearden et al., 2002; Droit-Volet et al., 2007). Ample evidence indicates that fear-provoking stimuli impair working memory (Kensinger and Corkin, 2003; Woodson et al., 2003). Thus, the unpredictable frightening stimuli likely damage the memory consolidation of the interval duration, resulting in the loss of pulses during the temporal representation process.

Experiment 1 showed that the fear-relevant stimuli induced shorter blank interval estimation compared to the neutral stimuli under unpredictable condition. According to prior studies, predictability modulated the susceptibility to fearful stimuli. Specifically, the predictable fear-relevant stimuli produced less emotional impact than unpredictable ones during the stimuli were presenting (Onoda et al., 2006; Yang et al., 2010, 2012). Furthermore, as we mentioned above, predictable fearful stimuli were found to lengthen the perceived duration of geometric shapes or blank interval (Droit-Volet et al., 2010; Yoshie and Haggard, 2013). However, the above two studies all used auditory stimuli. To test whether the predictability of fearful stimuli also influence the subjective experiences of time in visual system and to compare the effect of unpredictable and predictable fearful stimuli on interval timing, we conducted another two experiments (Experiments 2a and 2b) in which the emotional outcomes were all predictable to investigate the effects of these fear-related stimuli on the temporal estimation of the blank interval.

## Experiment 2

### Methods

#### Participants

A new sample of 34 right-handed college students (mean age = 19.91 years, *SD* = 1.65, including 4 males) participated in Experiment 2a. 20 right-handed college students (mean age = 23.05 years, *SD* = 2.14, including 5 males) participated in Experiment 2b. All participants were right-handed with normal or corrected-to-normal visual acuity. Informed consent was obtained from all participants. After the experiment, the participants were paid for their participation. These experiments were approved by the Ethics Committee of the Institute of Psychology, Chinese Academy of Sciences.

#### Materials

The apparatus and visual stimuli used in Experiments 2a and 2b were the same as those used in Experiment 1a.

#### Procedure

The procedures used were similar to those used in Experiment 1a with two exceptions. First, during the main test phase in Experiment 2a, a block design was used, in which three valences of the IAPS pictures presented separately. A block contained only one of the three valences of emotional or neutral pictures, such as fearful, neutral or happy. Besides, the trials with gray square were canceled. This design made the valence of each trial predicable in advance in a block. Second, the interval duration was analyzed as an independent variable in Experiment 2a. Thus, the blank interval between the offset of the fixation and onset of an images were selected from two different durations, 400 and 800 ms, instead of original 610 and 640 ms. The participants completed three blocks of 48 trials (8 × 2 × 3), 8 for each picture of fearful, neutral or happy IAPS images, 2 for each interval duration, and 3 for the occurrence number. This process resulted in a total of 144 trials. The order of the three blocks and the trials within each block were all randomized. The participants were allowed to take a break between each block. The design of trial sequences in Experiment 2a is illustrated in **Figure 1B**.

In Experiment 2b, a cueing paradigm was used to generate more explicit anticipation. To reduce the interaction of different valences of pictures, a modified oddball paradigm was used. In about 70% of the trials (48 trials in each block), an IAPS image was shown, and in the other 30% of trials (20 trials in each block), a gray square was presented. When a gray square was presented, the participants were told they did not need to estimate the interval duration as did in Experiment 1a. Example trial sequences in Experiment 2b are shown in **Figure 1C**. During each trial, the participants observed an initial cue (happy, unhappy, or neutral smiley or a yellow circle) for 300 ms, indicating that the outcome could be happy, fearful or neutral (i.e., happy, fearful, or neutral image) or a gray square, which was followed by a cross-fixation. The interval between cue and cross-fixation was 500–700 ms randomly. The following procedure was similar to that used in Experiment 2a, except for that all three emotional valence images were presented randomly in a block. The experimental phase consisted of 204 trials divided into three blocks of 68 trials each. The participants were allowed a short rest after each block. After the experiment, the participants were asked whether they understood and used the cue to anticipate the next stimulus.

In this experiment, the participants were required to complete a basic time discrimination test before the experiment as described in Experiments 1a and 1b.

### Results

A 2 (interval duration) × 3 (valence) within-subjects analysis of variance (ANOVA) was performed to analyze the subjective perception of the interval duration. In Experiment 2a, the ANOVA revealed a main effect of emotional valence, *F*(2, 66) = 4.07, *p* < 0.05, $\eta_{p}^{2}$ = 0.11; a main effect of interval duration, *F*(1, 33) = 148.00, *p* < 0.001, $\eta_{p}^{2}$ = 0.82; and no interaction between emotional valence and interval duration, *F*(2, 66) = 0.72, *p* = 0.49. The mean subjective perceived interval duration for each emotional valence is plotted in **Figure 2B**. According to the *post hoc* tests (Bonferroni), the mean reported time of the perceived interval of fearful pictures (*M* = 513.26, *SD* = 190.64) was greater than that of the neutral pictures (*M* = 476.88, *SD* = 189.90), and this difference reached significance, *p =* 0.02, whereas the perceived interval difference between the happy and neutral pictures was not significant, *p =* 0.07. In addition, no significant difference was observed between the fearful and happy pictures, *p =* 0.36.

In Experiment 2b, the results revealed a main effect of interval duration, *F*(1, 19) = 151.23, *p* < 0.001, $\eta_{p}^{2}$ = 0.89, and a main effect of emotional valence, *F*(2, 38) = 6.37, *p* < 0.01, $\eta_{p}^{2}$ = 0.25. The interval duration × valence interaction was not significant, *F*(2, 38) = 0.65, *p* = 0.53. According to the *post hoc* tests (Bonferroni), the perceived interval in the fearful trials was estimated to be significantly longer than that in the neutral trials, *p* < 0.01, but the difference the between happy and neutral trials was not significant, *p* = 0.45. In addition, no significant difference was observed between the fearful and happy trials, *p* = 0.19 (see **Figure 2C**).

### Discussion

The results of Experiments 2a and 2b provided convergent evidence that under predictable emotional outcome conditions, the interval between action and the predictable fearful picture was judged as being longer than that between action and the predictable neutral picture.

Experiencing fear has been shown to lengthen the duration in the presence of the frightening stimulus (Tipples, 2011; Fayolle et al., 2015; Eberhardt et al., 2016). However, the frightening stimulus did not actually appear during the clock stage in the current study. Furthermore, why was the blank interval before the predictable fear-relevant stimulus still prolonged? Arousal has been suggested to play a major role in emotional time distortions (Mella et al., 2011; Gil and Droit-Volet, 2012; Van Volkinburg and Balsam, 2014; Yoo and Lee, 2015). As arousal increases, the speed of the inner clock accelerates, resulting in a longer estimation for the same period of duration. In a predictable fearful situation, people tend to overestimate the degree of fear to account for the fear-relevant lengthening effect. This strong tendency to over-predict fear is considered related to other types of psychological overestimation (Rachman, 1994). Indeed, the ratings of the SAM scale in our material assessment revealed that the self-reported arousal level in response fearful pictures was significantly higher than that in response to happy pictures. Accordingly, for the predictable fearful outcomes, the lengthening effect may be induced by the acceleration of the clock speed. The participants may enter a “preparation status” before the fearful pictures actually emerge. The arousal level increased in the presence of the fear cue (for Experiment 2b) or during the entire block of fearful condition (for Experiment 2a), and the interval between the action and fearful picture was overestimated due to an arousal-induced acceleration of the clock mechanism. As suggested in a previous study investigating fear induced by electric shock, the aversive stimulus automatically activated a series of physiological responses associated with fear, such as increased heart rate, dilated pupils, and contracted muscles, thereby accelerating the internal clock (Fayolle et al., 2015). This explanation was supported by another study that used a predictable threatening event as the stimulus to explore the effect on time perception (Droit-Volet et al., 2010). In our study, the anticipation of a forthcoming fear-inducing stimulus could also increase the level of arousal similarly to an experienced fearful stimulus.

## General Discussion

The present study aimed to investigate the effects of predictable and unpredictable emotional stimuli on the perceived interval duration. Thus, we manipulated the predictability of the valence of the emotional outcome across several different temporal tasks, including modified verbal estimation and temporal bisection paradigm. Our findings revealed that the interval between a key-press action and an unpredictable or a predictable fearful picture, compared to a neutral picture, led to a reversed time illusion. We observed that the interval was underestimated under the unpredictable fear condition compared to the neutral condition; in contrast, the interval was overestimated under the predictable fearful condition compared to the neutral condition. The current findings are consistent with previous studies that have reported the lengthening effect of negative stimuli (Droit-Volet et al., 2010; Yoshie and Haggard, 2013). However, the present findings expand upon previous studies by revealing that unpredictable fear had a reverse effect on individuals’ interval estimation. Therefore, these effects can be interpreted as reflecting the unique mechanism of expectancy of fear-relevant stimuli on temporal perception.

According to the scalar expectancy theory, the timing process consists of the following three main stages: clock, memory, and decision-making. During the clock stage, which is also known as the prominent PA model of timing, two factors have been reported to affect individuals’ estimation of time, i.e., arousal and attention (Droit-Volet and Meck, 2007; Lake et al., 2016). We observed a temporal overestimation of predictable fear-relevant stimuli in both Experiments 2a and 2b. The observed temporal overestimation could be explained as arousal modulating the speed of a pacemaker, even though the fear was not actually present during the blank interval. According to the PA model of timing, the duration of a particular interval can be represented by the number of pulses collected by the accumulator. The overestimation or underestimation of duration may be due to changes in the rate of the pacemaker or the opening or closing latency of the switch (Gibbon et al., 1984; Church, 2002). The official ratings and our ratings of the pictures used showed that the fearful pictures were more arousing than the happy pictures used in our study. Therefore, our results support the hypothesis that predictable fear increases arousal during the blank interval and, consequently, the rate of the pacemaker, leading to an overestimation of the interval. However, another study provided an additional explanation from the perspective of the sense of agency, suggesting that due to self-serving biases, people were unwilling to take responsibility for unpleasant outcomes and thus distance themselves from voluntary actions leading to negative outcomes (Yoshie and Haggard, 2013).

However, the shorter subjective experience of the blank interval between an action and unpredictable emotional outcome requires further discussion. During the clock stage, the three conditions likely do not differ. The fear-relevant stimuli were effective in capturing attention (Öhman et al., 2001; Georgiou et al., 2005; Fox et al., 2007). The unpredictable fearful picture automatically attracted more exogenous attention than the neutral and happy pictures. Our findings support the hypothesis that the time distortion is a complex interaction between arousal and cognitively driven processes involved in directing resource-sharing of attention resources between emotional and timing processes. Nevertheless, under the unpredictable condition, the attention allocation during the blank interval was the same in all three valence trials. However, the fear-relevant stimuli were effective in automatically capturing attention (Öhman et al., 2001; Georgiou et al., 2005; Fox et al., 2007). Therefore, compared to neutral or happy pictures, unpredictable fearful stimuli, result in more attention diverted to the stimuli themselves other than to keeping the perceived temporal information in mind, thus disrupting temporal memory consolidation. Because an unexpected stimulus attracts more attention than an expected stimulus (Ulrich et al., 2006; Summerfield and Egner, 2009), a fearful picture under the unpredictable condition should distract more attention from memory consolidation than that under the predictable condition, resulting in a higher number of missed registered pulses and a shortening effect due to unpredictable fear.

**FIGURE 3:**
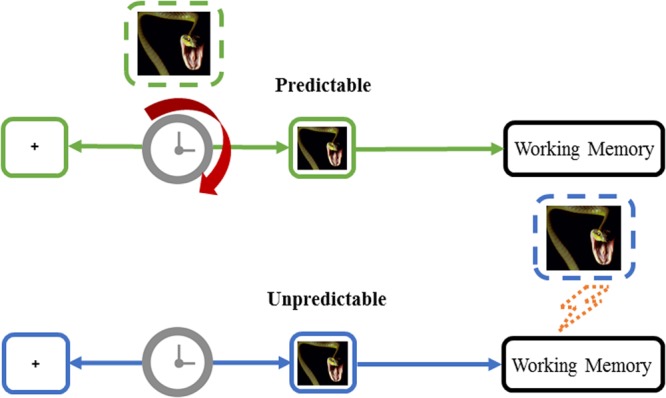
A schematic illustration of the interval temporal distortion processes under the unpredictable and predictable fear-relevant stimulus conditions. Colored boxes and arrows indicate the unpredictable (blue) and predictable (green) conditions. The green dotted box represents the expected fear that accelerates the internal clock during the blank interval. The blue dotted box represents that the unpredictable fear-relevant stimuli disturb the working memory stage and result in reduced temporal estimation.

As mentioned above, memory is a key stage of temporal perception and judgment (Matthews and Meck, 2016). In prospective temporal tasks, such as those performed in our experiments, time judgments depend on comparisons between the target duration and previously encoded intervals (Matell and Meck, 2004). In our study, the unpredictable fearful condition may interrupt the memory representation of the estimated blank interval before the picture. Because all three valence pictures were unpredictable, the clock stages of the blank intervals for all three valences were the same until the picture was presented. Therefore, the memory stage of the inner clock model may be critical for the discrepancy among the different valence pictures under the unpredictable condition. Many studies had demonstrated that fearful stimuli could impair working memory (Kensinger and Corkin, 2003; Woodson et al., 2003). In addition, evidences showed that emotional expectations modulated the neural sensitivity to emotional stimuli. And unpredictable emotional stimuli produced greater impact on neural processing (Onoda et al., 2006; Yang et al., 2010, 2012). Furthermore, according to the Decision Affect Theory (DAT), unpredictable bad outcomes feel worse than predictable bad outcomes (Shepperd and McNulty, 2002). Thus, the unpredictable fear in our study may cause much more serious damage to working memory than predictable fear, leading to an underestimation of the blank interval relative to that under predictable condition. The processes of interval temporal distortion under predictable and unpredictable fearful conditions could be explained in **Figure 3**.

An alternative explanation should also be mentioned. Fear is a biologically relevant adaptive emotion that plays a critical role in human survival. Human beings evolved in environments in which resources and dangers were unpredictably distributed in space and time (Öhman et al., 2001). Rapidly detecting the unpredictable dangers can increase the chances of survival. For example, a snake in the grass will be found more quickly than a fear-irrelevant stimulus (Öhman et al., 2001). The processing of fear-relevant stimuli is prioritized. Accordingly, time distortions may reflect the flexibility of the neural timing system and thus allow organisms to adapt to their environment (Droit-Volet and Gil, 2009; Droit-Volet et al., 2010). In our study, the shortening effect of the unpredictable fear-relevant stimuli may reflect a perceived increase in the processing of a threatening object. Such an effect is adaptive because it could result in the execution of a behavioral reaction earlier to avoid dangerous stimuli.

Our present study also has some limitations. First, the specificity of IAPS pictures for fear and happiness were not appraised based on our subjects’ own experiences. Although we selected the materials based on the previous research about the category data of the IAPS and re-rated the arousal and valence of the pictures that we used in our own sample, the individual differences in perceiving the fearful and happy IAPS pictures are still difficult to eliminate. Second, to manipulate the predictability of the emotional stimuli, different paradigms were employed in our experiments. To realize the unpredictable condition, we randomized the emotional stimuli in each block in Experiments 1a and 1b; whilst predictable condition was realized by a block design and a cueing design. The differences in the paradigms may induce confounding to isolate the factor of anticipation. In future studies, the predictability of fear-relevant stimuli on interval timing could be further investigated by manipulating the probabilities of the presentation of different emotional stimuli in one experiment.

## Conclusion

The present study provided preliminary evidence that the modulating effect of emotion on time perception may work at different stages. Even in the absence of fearful stimuli, the time perception of the blank interval before the fear-relevant pictures varied depending on the predictability. In addition, the unpredictability of fearful outcomes might affect time perception during the memory stage rather than during the clock stage.

## Author Contributions

QC, KZ and XF contributed to designing the experiments, analyzing the data, and writing the manuscript. QC contributed to collecting the data. Y-HC and WZ contributed to writing the manuscript.

## Conflict of Interest Statement

The authors declare that the research was conducted in the absence of any commercial or financial relationships that could be construed as a potential conflict of interest.
